# Hyper-oncotic albumin administration reduces mortality in acute Respiratory Distress Syndrome compared to crystalloid: a systematic review and meta-analysis

**DOI:** 10.1080/07853890.2026.2637271

**Published:** 2026-03-24

**Authors:** Yan-Jie Zhang, Zhu Luo, Zhen Wang, Ji-Yue Shao, Jingyuan Xu

**Affiliations:** ^a^ Department of Critical Care Medicine, Zhongda Hospital, School of Medicine, Southeast University, Nanjing, Jiangsu, P. R. China; ^b^ Jiangsu Provincial Key Laboratory of Critical Care Medicine, Department of Critical Care Medicine, Zhongda Hospital, School of Medicine, Southeast University, Nanjing, P. R. China

**Keywords:** Acute Respiratory Distress Syndrome (ARDS), albumin, hyper-oncotic albumin, fluid management, mortality

## Abstract

**Background:**

To evaluate the association between albumin administration as volume replacement and mortality in adult ARDS patients, we performed this meta-analysis and trial sequential analysis (TSA).

**Methods:**

We searched databases including PubMed, Science Direct, Scopus, Web of Science databases and Cochrane Central Register of Controlled Trials up to 12 December 2024. We screened trials that included adult ARDS patients and compared albumin with crystalloid. The 28-day mortality served as the primary endpoint, while the oxygenation change, the length of ICU stay and the length of hospital stay were designated as secondary outcomes. To clarify the differing concentrations of albumin, we formed two distinct subgroups: the hyper-oncotic albumin subgroup (≥20%) and the iso-oncotic albumin subgroup (4%∼5%). Statistical synthesis was performed with Cochrane Review Manager 5.4.1, employing random-effects models. To mitigate random errors, TSA was implemented with α = 0.05 and β = 0.20 parameters.

**Results:**

The analysis incorporated 5 publications: 3 randomized controlled trials (RCTs) and 2 non-randomized studies (NRSs). Overall mortality was lower in the albumin group (33.2%, 97/292) than in the crystalloid group (44.9%, 133/296) (OR = 0.61, 95%CI 0.43–0.85, *p* = 0.004). RCTs (*n* = 204) showed no benefit (OR = 0.83, *p* = 0.54), but NRSs (*n* = 384) demonstrated reduced mortality (OR = 0.52, *p* = 0.002). Hyper-oncotic albumin was associated with lower mortality in NRSs (OR = 0.40, *p* = 0.02) but not in RCTs (OR = 0.74, *p* = 0.57). Iso-oncotic albumin showed no benefit (OR = 0.88, *p* = 0.72). Regarding the impact of albumin on oxygenation, significant improvements in oxygenation were observed only on the first (*p* = 0.05) and second days (*p* < 0.0001). The TSA indicated a continued need for high-quality RCTs.

**Conclusions:**

Our analysis suggests that hyper-oncotic albumin may reduce mortality and improve early oxygenation in ARDS patients compared to crystalloids. Larger RCTs are urgently needed to validate these findings and define their potential role in clinical management.

## 1. Background

Acute respiratory distress syndrome (ARDS), common in critical care, stems from pulmonary inflammation that disrupts the alveolar–capillary barrier, causing edema and compromised gas exchange with consequent hypoxemia [1]. ARDS is associated with significant rates of mortality [2], which pose a substantial challenge to public health [3]. Despite the advances in our understanding of ARDS in recent years, treatment options remain primarily supportive.

Fluid management is one of the major treatment modalities for ARDS [4]. The conservative fluid management strategy is considered by the Fluid and Catheter Treatment Trial (FACTT) study to improve the oxygenation index [5] and a recent meta-analysis indicated that restrictive fluid resuscitation may reduce the duration of mechanical ventilation [6].

Albumin, as the primary component of plasma proteins, not only improves plasma oncotic pressure, reduces tissue edema, and increases blood volume [7]. It has also been investigated in conjunction with diuretics with the aim of enhancing fluid removal and improving pulmonary edema. While some evidence suggests such combination can lead to short-term improvements in oxygenation and fluid balance, it is noted that these effects may be transient and the overall evidence remains limited [8]. Consequently, albumin use in patients with ARDS may play a pivotal role in conservative fluid management strategy, which may lead to an increase in the patient’s oxygenation index and an improved prognosis [9].

In the study by Philipis et al., the use of albumin demonstrated an improvement in hemodynamics and survival rates [10]. An animal study also demonstrated that albumin solution is associated with a reduced pulmonary injury [11]. However, several randomized controlled trials (RCTs) conducted in patients with ARDS have not yielded positive results in terms of short-term mortality [12–14].

Recent comprehensive guidelines for ARDS management appropriately focus on definitions, phenotyping, and ventilatory support, and thus do not extend recommendations to fluid balance or albumin use [15,16]. Guidelines dedicated to fluid therapy in critical illness evaluate albumin but underscore the persisting uncertainty of the evidence [17]. Furthermore, expert consensus specifically on albumin use in critically ill patients acknowledges its potential physiological rationale while highlighting the need for more robust clinical data, particularly in specific subpopulations like ARDS [18].

Albumin, based on its concentration, can be classified into hyper-oncotic albumin (with concentrations of 20% or higher) and iso-oncotic albumin (with concentrations of 4 ∼ 5%). Hyper-oncotic albumin, due to its higher oncotic pressure, is more effective in increasing plasma oncotic pressure, reducing extravasation of fluid from the vasculature, while iso-oncotic albumin is primarily used to supplement blood volume [19,20]. Although hyper-oncotic albumin has shown efficacy in the field of critical cranial trauma compared to iso-oncotic albumin [21], there is a lack of related research that analyses its use in the context of ARDS.

This meta-analysis was undertaken to evaluate the association between albumin administration, especially different concentration of albumin as volume replacement and mortality in adult patients with ARDS.

## 2. Methods

We adhered to the PRISMA 2020 statement for reporting systematic reviews and meta-analyses (Supplementary Materials 1) and its abstract (Supplementary Materials 2) [22]. A protocol was lodged with PROSPERO database (CRD42024599118).

### 2.1. Search strategy

Database including PubMed, Science Direct, Scopus, Web of Science databases and Cochrane Central Register of Controlled Trials. The search combined the following key terms.: ((albumin) OR (human serum albumin) OR (human albumin) OR (albumin replacement) OR (colloid) OR (crystalloid) OR (crystalloid solution)) AND ((acute respiratory distress syndrome) OR (ARDS) OR (acute lung injury) OR (ALI) OR (critical ill) OR (intensive care unit) OR (critical care) OR (ICU) OR (intensive care)) AND ((randomized controlled trial) OR (controlled clinical trial) OR (placebo) OR (retrospective) OR (random* AND (trial OR study OR group)) OR ((controlled OR comparative) AND (trial OR study))). Complete search strings are provided in Additional file 1. The query was run without language limits across all listed databases from inception to 12 December 2024; supplementary materials of retrieved articles were like-wise screened. The detailed search strategy for all databases is provided in Supplementary Materials 3.

### 2.2. Study selection

A single reviewer first examined the full texts of all potentially relevant records identified by the search. Afterward, the same set of manuscripts was independently assessed against the inclusion criteria by two reviewers. Disagreements between the two reviewers were reconciled through consensus or, when needed, by consultation with a third reviewer. Whenever duplication – whether authorship or dataset – was suspected, the latest publication was retained.

#### 2.2.1. Inclusion criteria

 

1. Randomized controlled trials and non-randomized studies (NRSs).
2. The study population exclusively consisted of adult patients with acute lung injury (ALI) [23] or acute respiratory distress syndrome (ARDS) [24] or reported data on a subgroup of patients with ALI/ARDS
3. Receive albumin administration as volume replacement.

#### 2.2.2. Excluded criteria

 

1. Studies that included patients with ALI/ARDS but did not report separate data for this population, making data extraction impossible.
2. No albumin was used as the fluid intervention in the trial group.
3. No crystalloid was used for comparison with albumin.
4. Data on mortality in patients were not included.
5. Full text articles were not available.

### 2.3. Data extraction and management

Two reviewers independently extracted data using a predefined extraction sheet. Discrepancies were settled through discussion with a third reviewer until consensus was reached. Another reviewer subsequently audited the extracted data for accuracy. Data reported solely in figures were digitized with Origin 2021 Pro (OriginLab, Northampton, MA, USA). Additional variables captured were design, number of centers, enrolment size, sex and age distributions, intervention details, outcome, and lengths of ICU and hospital stay.

### 2.4. The risk of bias

For different types of studies, we employ different methodologies for quality assessment. The Risk of Bias 2 (ROB2) [25] was used to assess the risk of bias for the RCT. The Risk of Bias in Non-randomized Studies-of Interventions (ROBINS-I) [26] was used to assess the risk of bias for NRS. The risk of bias of each article was independently assessed by two reviewers. Disagreements between the two reviewers were reconciled through consensus or, when needed, by consultation with a third reviewer.

### 2.5. Outcome measures and subgroup analysis

The primary outcome was 28-day mortality, while the oxygenation change, the length of ICU stay and the length of hospital stay were designed secondary outcomes.

To further investigate the effect of albumin on mortality in patients with ARDS, we performed subgroup analyses based on the albumin concentration used. Specifically, we categorised the studies into the following two subgroups: (1) hyper-oncotic albumin subgroup: studies employing albumin with a concentration of 20% or higher were included in this subgroup. (2) Iso-oncotic Albumin Subgroup: Studies employing albumin with a concentration of 4 ∼ 5% were included in this subgroup. Through this analysis, we compared the efficacy of albumin therapy with crystalloid therapy in each subgroup, seeking to identify the possible effects of varying albumin concentrations on mortality rates among ARDS patients.

### 2.6. Assessment of the certainty of the evidence

To assess the certainty of each study and the evidence for the outcomes listed above, the Grading of Recommendations Assessment, Development and Evaluation (GRADE) system was used [27]. The quality of each article was independently assessed by two reviewers. Disagreements between the two reviewers were reconciled through consensus or, when needed, by consultation with a third reviewer.

### 2.7. Assessment of heterogeneity and publication biases

Inter-study heterogeneity was gauged with I^2^ statistics, estimating the percentage of total variability attributable to true differences rather than chance. Heterogeneity was categorized: I^2^<25% (none), 25–50% (low), 50–74.9%(moderate) and 75–100%(high) [28].

To visually assess publication bias, a funnel plot was constructed and the Egger regression test was performed to quantitatively evaluate the asymmetry of the funnel plot.

### 2.8. Trial sequential analysis

To mitigate the risk of type-I error inflation due to repeated significance testing and to integrate sample size estimation with an adjusted significance level in our meta-analysis, trial sequential analysis (TSA; Copenhagen Trial Unit, Denmark, version 0.9 Beta) was utilized. This analysis used O’Brien-Fleming boundaries and determined the diversity-adjusted information size required to detect a prespecified 20% relative risk reduction (α = 0.05, power = 80%).

### 2.9. Statistical analysis

Review Manager 5.4.1 (The Nordic Cochrane Centre, Rigshospitalet, Copenhagen, Denmark) and R version 4.4.1 (R Foundation for Statistical Computing, Vienna, Austria) were used to assess the data.

The odds ratio (OR) for dichotomous data and weighted mean differences (WMD) for continuous data were estimated, with 95% confidence intervals (CI). Continuous variables following normal distributions were summarized as mean with standard deviation (SD). Non-normal data were expressed as medians with interquartile ranges (IQR; reported as P_25_–P_75_). Categorical outcomes were presented using frequencies and percentages. To ensure data uniformity, Wan’s method converted medians to mean and SD estimates *via* the formula SD ≈ IQR/1.35 [29]. Statistical significance was defined by a two-sided α-level of 0.05.

Taking into account the design heterogeneity between RCTs and NRS, the inverse variance method was employed for the meta-analysis. This approach weights the studies on the basis of their variances, providing a more robust and reliable synthesis of the evidence. The random-effects model was used due to its ability to more effectively manage heterogeneity among studies, thus mitigating biases attributable to differences in study designs and providing more reliable and accurate pooled results [30]. Sensitivity analyses were undertaken to explore sources of substantial heterogeneity.

## 3. Results

### 3.1. Study selection

**Figure 1.**
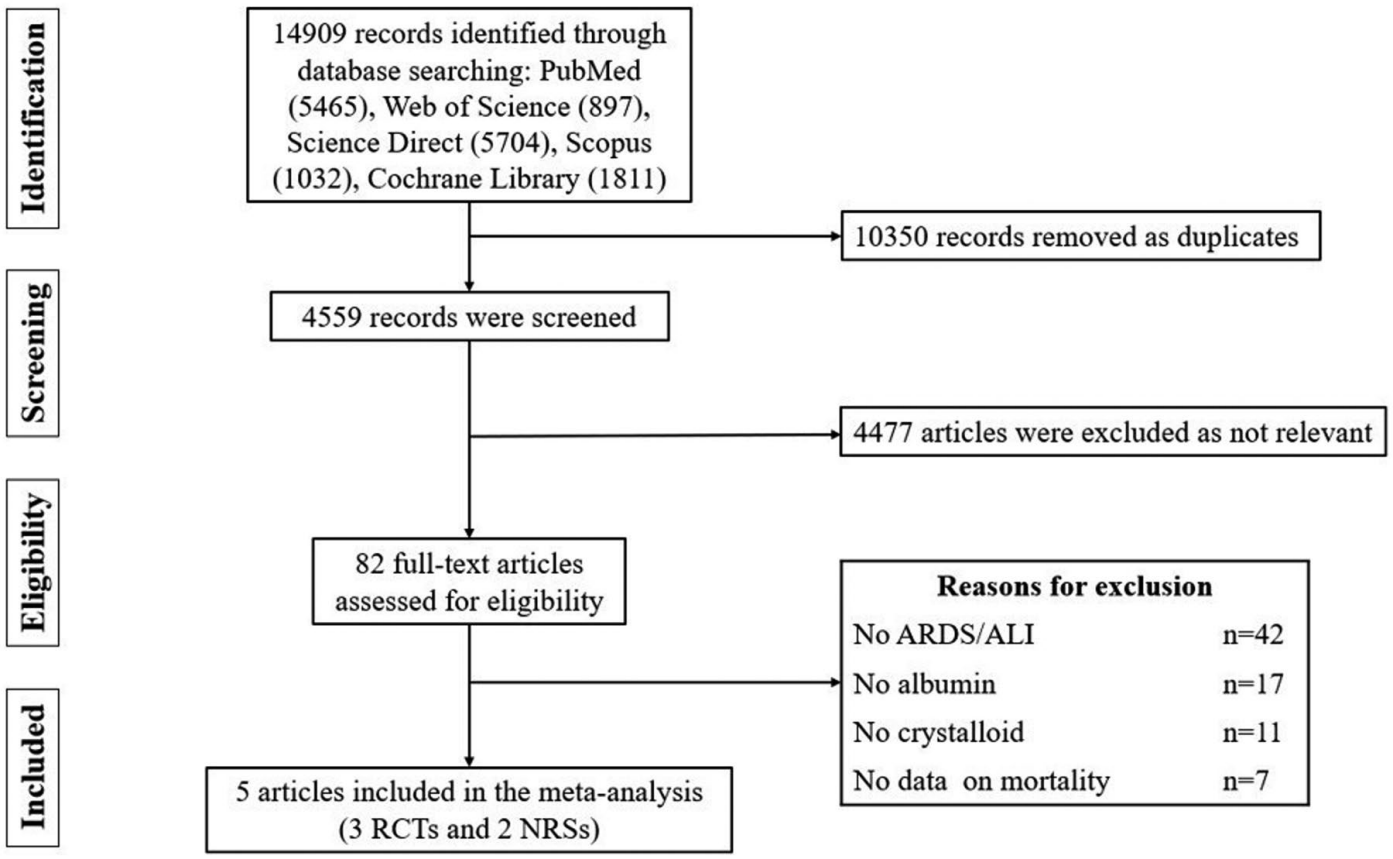
Flow diagram of the databases search.

The literature search initially retrieved 14,909 records. Following duplicate removal (*n* = 10,350), 4,559 unique publications underwent title/abstract screening. This process excluded 4,477 articles. Figure 1 details the study selection flowchart. Among 82 potentially eligible studies, 77 were subsequently excluded. All exclusion rationales and study characteristics can be seen in Supplementary Materials 4. Consequently, five trials [12–14,31,32] met inclusion criteria.

### 3.2. Study characteristics

Table 1 summarizes characteristics of included trials and their comparative primary outcomes. The meta-analysis encompassed 588 ARDS cases, with 292 assigned to albumin administration versus 296 instead receiving crystalloid. Three of the trials [12–14] were RCTs, while one of the trials [31] was a case-control study and one of the trials [32] was a cohort study. Four trials [12–14,31] involved patients with ALI, while the rest [32] enrolled patients with ARDS. In three trials [12,14,31], albumin was administered in conjunction with furosemide. Among the studies, three [12,14,31] used hyper-oncotic albumin (with concentrations of 20% or greater), one [13] used an iso-oncotic (with concentrations of 4 ∼ 5%), and in one study [32], the colloid osmotic pressure of albumin was not specified.

**Table 1.** General characteristics of the studies included.

Author year	Study design	Population (sample size)	Sample size	Key intervention & control		Primary goal of the intervention	Key point on heterogeneity
				Intervention	Control		
Martin 2002 [12]	Double-blind, placebo-controlled RCT	ALI patients with hypoproteinemia (total protein ≤5.0 g/dL)	19/18	25% hyperoncotic albumin + furosemide	Placebo (normal saline)	To improve physiological parameters (oxygenation, fluid balance) in hypoproteinemic ALI patients.	Goal: Tests a physiological treatment strategy (albumin + diuretic) in a specific phenotype (hypoproteinemia).
Finfer 2004 [13]	Multicenter, double-blind, pragmatic RCT	General ICU patients requiring fluid resuscitation (ARDS as a subgroup)	61/66	4% iso-oncotic albumin for all resuscitation	Normal saline for all resuscitation	To compare the effect of two resuscitation fluids on 28-day mortality in a general critically ill population.	Context: Addresses general fluid resuscitation using iso-oncotic albumin. ARDS outcome is a post-hoc subgroup analysis.
Martin 2005 [14]	Double-blind, placebo-controlled RCT	ALI/ARDS patients with hypoproteinemia (total protein <6.0 g/dL)	20/20	25% hyperoncotic albumin + furosemide	Placebo (normal saline) + furosemide	To determine the additive benefit of albumin on top of furosemide for improving oxygenation/fluid balance.	Goal: Factorial design aiming to isolate the independent contribution of albumin within a combined therapy.
Cordemans 2012 [31]	Retrospective, matched case-control	ALI patients at risk for intra-abdominal hypertension	57/57	PAL protocol (high PEEP + hyperoncotic albumin for small-volume resuscitation + furosemide/UF)	Usual care patients matched from a historical cohort	To evaluate a multi-modal, restrictive fluid strategy aimed at achieving negative balance and reducing IAH/pulmonary edema.	Design & Intervention: Complex, multi-component intervention; uses historical controls (non-randomized).
Wang 2023 [32]	Retrospective cohort study (MIMIC-III database)	Septic shock patients with ARDS	135/135	Early albumin administration (any concentration/dose) within 24h of ICU admission	Propensity-score matched patients without early albumin use	To explore the observational association between early timing of albumin administration and outcomes in a real-world setting.	Design & Exposure: Purely observational; the exposure is “early use” (timing), not a standardized treatment protocol.

* RCT: randomized controlled trial; ALI: acute lung injury; ICU: intensive care unit; ARDS: acute respiratory distress syndrome.

The ROB 2 evaluation for RCTs (Table 2) indicated that Finfer 2004 [13] was rated as low risk in all domains (randomisation process, deviations from intended interventions, missing outcome data, measurement of outcome and selection of reported results), with an overall low risk of bias. However, the studies by Martin 2002 [12] and Martin 2005 [14] had some flaws in the randomization process (due to the lack of a detailed description of allocation concealment) and in the selection of reported results (no mention of study registration or predefined outcomes), leading to an overall bias rating of “some concerns.”

**Table 2.** Results of the Cochrane risk of bias 2 tool for randomized clinical trial.

Author year	Randomization process	Deviations from intended interventions	Missing outcome data	Measurement of the outcome	Selection of the reported results	Overall bias
Martin 2002 [12]	Some concerns*	Low	Low	Low	Some concerns**	Some concerns
Finfer 2004 [13]	Low	Low	Low	Low	Low	Low
Martin 2005 [14]	Low	Low	Low	Low	Some concerns**	Some concerns

* The randomization method did not provide a detailed description of allocation concealment (only mentioning a “computer - generated list”).

** There was no mention of study registration or predefined outcomes.

For NRS (Table 3), the ROBINS-I tool assessment revealed that Cordemans 2012 [31] had a moderate overall risk of bias and Wang 2023 [32] had a high overall risk of bias. Cordemans’ study [31] was marked as moderate risk due to incomplete control of all key confounder variables (although propensity score matching was used) and the potential inconsistency of interventions resulting from the retrospective design. Wang’s study [32] also suffered from bias in the classification of interventions (failure to distinguish the impact of different albumin concentrations). Both studies were considered low risk in other domains, such as participant selection, missing data, and outcome measurement. This identified risk of bias suggests a potential for overestimation of the treatment effect in these studies, a concern that is explored in the discussion of the mortality results.

**Table 3.** Results of the risk of bias in non-randomized studies-of interventions for non-randomized studies.

Author year	Bias due to confounding	Bias in selecting of participants into the study	Bias in classification of interventions	Bias due to deviations from intended interventions	Bias due to missing data	Bias in measurement of outcomes	Bias in selection of the reported result	Overall bias
Cordemans 2012 [31]	Moderate^a^	Low	Low	Moderate^d^	Low	Low	Low	Moderate
Wang 2023 [32]	Moderate^a^	High^b^	Moderate^c^	Moderate^d^	Low	Low	Low	High

^a^ Although propensity score matching was employed, the study did not explicitly state whether all key prognostic variables were controlled for.

^b^ Retrospective enrollment of participants.

^c^ The impact of albumin at different concentrations (such as 4% vs. 20%) was not distinguished.

^d^ The retrospective design cannot ensure consistency between the intervention and control groups during the treatment process, and the control group may have received other unrecorded interventions.

The studies by Martin 2002 [12] and Martin 2005 [14] were rated as moderate - certainty evidence due to their small sample sizes. In contrast, the study by Finfer 2004 [13] was rated as high – certainty evidence. Studies by Cordemans 2012 [31] and Wang 2023 [32], due to their non – randomized controlled design, were rated as low – certainty evidence (Table 4).

**Table 4.** The certainty of the evidence in each RCTs and NRSs.

Author year	Study design	Risk of bias	Inconsistency	Indirectness	Imprecision	Other consideration	Certainty
Martin 2002 [12]	RCT	Not serious	Not serious	Not serious	Not serious	Small sample size	Moderate
Finfer 2004 [13]	RCT	Not serious	Not serious	Not serious	Not serious	–	High
Martin 2005 [14]	RCT	Not serious	Not serious	Not serious	Not serious	Small sample size	Moderate
Cordemans 2012 [31]	Case-control	Not serious	Not serious	Not serious	Not serious	–	Low
Wang 2023 [32]	Cohort	Not serious	Not serious	Not serious	Not serious	–	Low

* RCT: Randomized controlled trial; NRS: non-randomized study.

** GRADE Working Group grades of evidence: High certainty: we are very confident that the true effect lies close to that of the estimate of effect. Moderate certainty: we are moderately confident in the effect estimate: the true effect is likely to be close to the estimate of the effect, but there is a possibility that it is substantially different. Low certainty: our confidence in the effect estimate is limited: the true effect may be substantially different from the estimate of the effect. Very low certainty: we have very little confidence in the effect estimate: the true effect is likely to be substantially different from the estimate of effect.

### 3.3. The impact of albumin on mortality in ARDS treatment

#### 3.3.1. Overall effects of albumin impact on mortality in ARDS treatment

**Figure 2.**
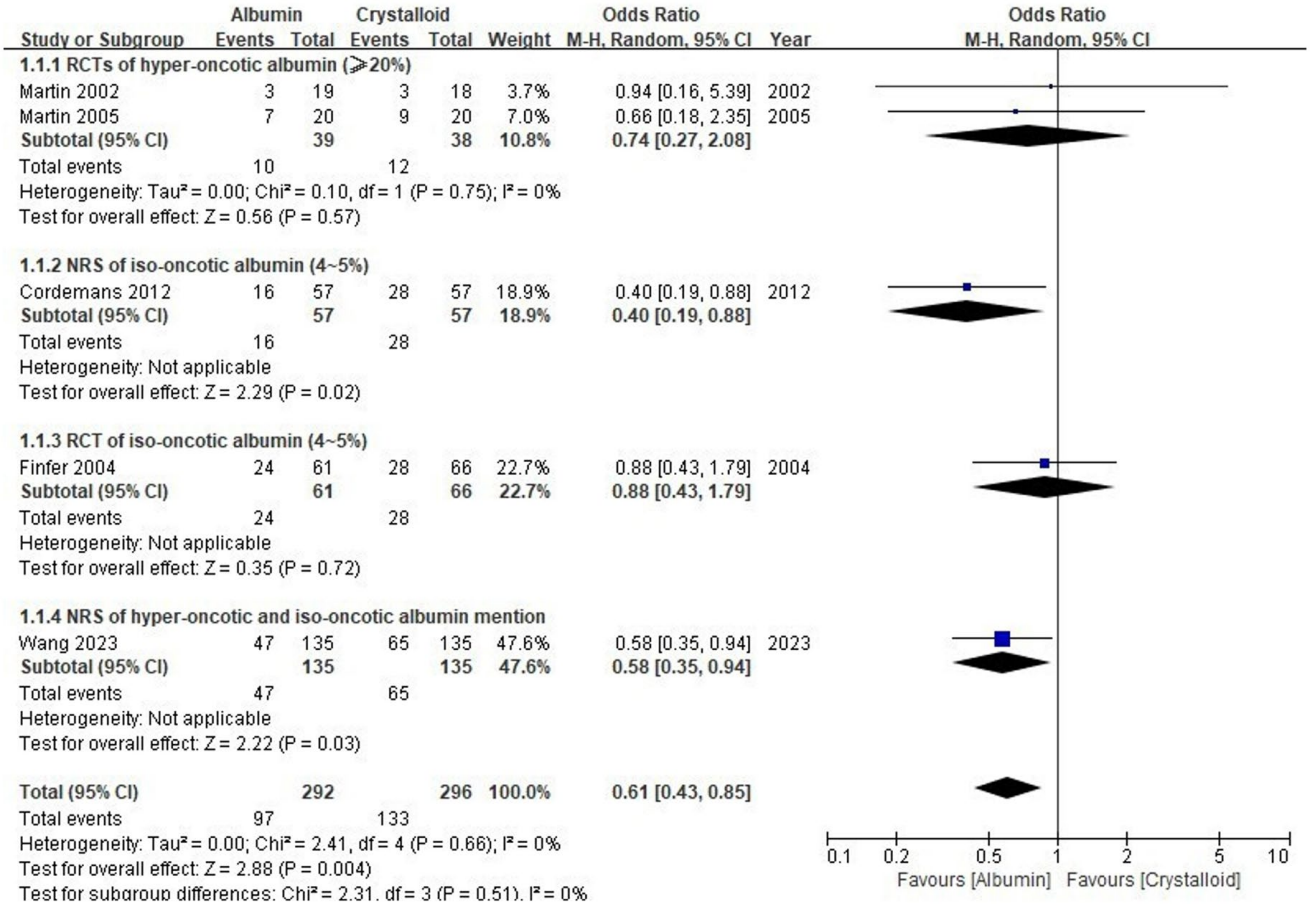
Forest plot of the association between albumin administration and mortality in patients with ARDS. Squares represent the point estimate of the odds ratio (OR) for each individual study, with the size of the square proportional to the study’s weight in the meta-analysis. Horizontal lines indicate the 95% confidence intervals (CI). The diamond represents the pooled OR and 95% CI for each subgroup and the overall analysis. The vertical line at OR = 1.0 denotes no effect. An OR < 1 favors the albumin group. The I^2^ statistic and *p*-value for heterogeneity are provided for each analysis. RCT: randomized controlled trial; NRS: non-randomized study.

The forest plot in Figure 2 illustrates the effects of albumin on mortality, with data derived from both RCTs and NRSs. The analysis includes a total of five trials and no significant heterogeneity was detected overall (*p* = 0.66, I^2^=0%). Combining both RCTs and NRSs, the overall mortality was 97 events out of 292 patients (33.2%) in the albumin group and 133 events out of 296 patients (44.9%) in the crystalloid group. The OR was 0.61 (95%CI: [0.43, 0.85], *p* = 0.004), indicating that albumin administration as volume replacement may reduce mortality in adult patients with ARDS.

A sensitivity analysis was conducted excluding Wang 2023 [31] due to its large sample size. This exclusion maintained a mortality reduction trend (*p* = 0.06) with I^2^=0%, as visualized in Supplementary Material 5 (Fig. S1).

#### 3.3.2. Analysis of RCTs on albumin impact on mortality in ARDS treatment

There were 3 RCTs (Martin 2002 [12], Finfer 2004 [13] and Martin 2005 [14]) with a total of 100 patients in the albumin group and 104 patients in the crystalloid group (Supplementary Material 5 Fig. S2). Overall mortality was 34 events out of 100 patients (34.0%) in the albumin group and 40 events out of 104 patients (38.5%) in the crystalloid group. The OR was 0.83 (95%CI: [0.47, 1.49], *p* = 0.54), indicating that albumin administration as volume replacement probably does not reduce mortality in adult patients with ARDS.

#### 3.3.3. Analysis of NRSs on albumin impact on mortality in ARDS treatment

There were 2 NRSs (Cordemans 2012 [31] and Wang 2023 [32]) with a total of 192 patients in each group. Overall mortality was 63 events out of 192 patients (32.8%) in the albumin group and 93 events out of 192 patients (48.4%) in the crystalloid group (Supplementary Material 5 Fig. S2). The OR was 0.52 (95%CI: [0.34, 0.79], *p* = 0.002), indicating that albumin administration as volume replacement may reduce the mortality in adult patients with ARDS.

#### 3.3.4. Analysis of the effect of albumin oncotic pressure on mortality in ARDS treatment

Two RCTs were included in the hyper-oncotic albumin subgroup (a concentration of 20% or higher), with a total of 39 patients in the hyper-oncotic albumin group and 38 patients in the crystalloid group (Figure 2). The hyper-oncotic albumin group had 10 events, and the crystalloid group had 12 events. The overall odds ratio was 0.74 (95% CI: [0.27, 2.08], *p* = 0.57).

One NRS was included in the hyper-oncotic albumin subgroup (a concentration of 20% or higher), with a total of 57 patients in the hyper-oncotic albumin group and 57 patients in the crystalloid group (Figure 2). The hyper-oncotic albumin group had 16 events, and the crystalloid group had 28 events. The overall odds ratio was 0.40 (95% CI: [0.19, 0.88], *p* = 0.02), indicating that hyper-oncotic albumin administration as volume replacement may potentially reduce the mortality in adult patients with ARDS.

There was only one trial included in the iso-oncotic albumin subgroup (Figure 2). The result showed that the administration of iso-oncotic albumin as volume replacement does not reduce mortality in adults with ARDS (OR: 0.88, 95%CI: [0.43, 1.79], *p* = 0.72).

The study of Wang did not distinguish between hyper-oncotic and iso-oncotic albumin, with a total of 135 patients in the albumin group and 135 patients in the crystalloid group (Figure 2). The albumin group had 47 events, and the crystalloid group had 65 events. The overall odds ratio was 0.58 (95% CI: [0.35, 0.94], *p* = 0.03)

**Figure 3.**
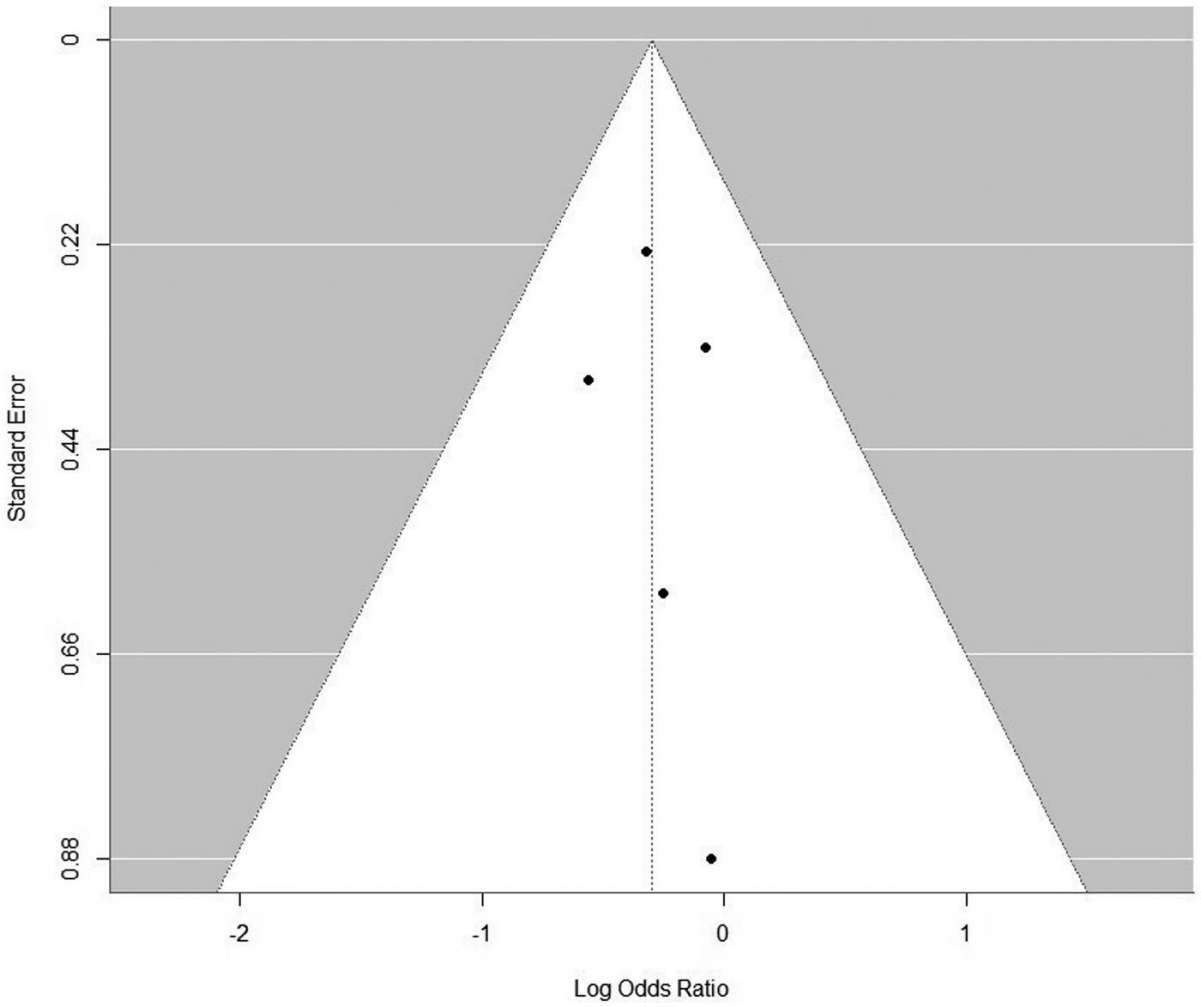
Funnel plot assessing publication bias for the primary outcome of mortality. Each point represents an individual study included in the meta-analysis, plotting its effect estimate (log odds ratio) against its precision (standard error). The vertical line indicates the overall pooled effect estimate. The dashed inverted funnel outlines the expected region within which 95% of studies should lie in the absence of bias and heterogeneity. Visual inspection suggests approximate symmetry, which was corroborated by Egger’s regression test (*p* = 0.8303). It is important to note that the power of these tests to detect asymmetry is limited when fewer than 10 studies are included.

Publication bias was assessed for the primary outcome through visual inspection of the funnel plot (Figure 3) and Egger’s regression test. The funnel plot showed an approximately symmetrical distribution of study points, and Egger’s test did not indicate significant asymmetry (*p* = 0.8303). However, the power of these tests to reliably detect bias is limited when the number of included studies is small (<10), and this possibility cannot be ruled out.

### 3.4. The effects of albumin on the oxygenation change in the treatment of ARDS

**Figure 4.**
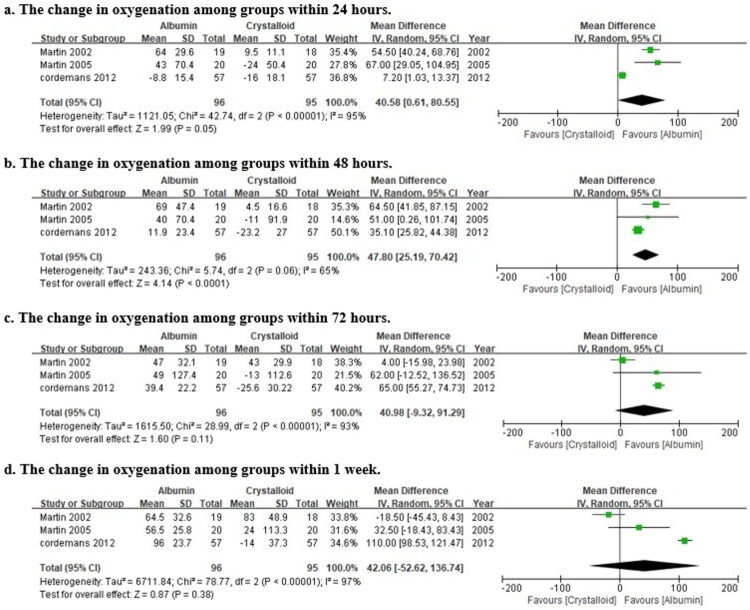
Forest plot of the weighted mean differences in the oxygenation index at different time points after albumin administration. Data are derived from 3 trials [12,14,31]. The squares represent the pooled weighted mean difference (WMD) for each time point, with the horizontal lines indicating the 95% confidence intervals (CI). The size of the square may reflect the weight or precision of each estimate. The vertical line at WMD = 0 indicates no difference. A WMD > 0 favors the albumin group (improved oxygenation). Significant improvements were observed at 24 and 48 h, but not at later time points.

The effects of albumin on the oxygenation change were calculated using data from 3 trials (Figure 4) [12,14,31]. Significant improvements in oxygenation were observed in the albumin group within 24 h (WMD = 40.58 mmHg, 95%CI: [0.61,80.55], *p* = 0.05) and 48 h (WMD = 47.80 mmHg, 95%CI: [25.19,70.42], *p* < 0.0001). However, these differences became less apparent at 72 h (WMD = 40.98 mmHg, 95%CI: [−9.32,91.29], *p* = 0.11) and at one week (WMD = 42.06 mmHg, 95%CI: [−52.62,136.74], *p* = 0.38).

The effects of albumin on the length of stay in the ICU and the length of hospital stay were not significantly different between the groups (Supplementary Material 5 Fig. S3 and Fig. S4).

### 3.5. Random errors

**Figure 5.**
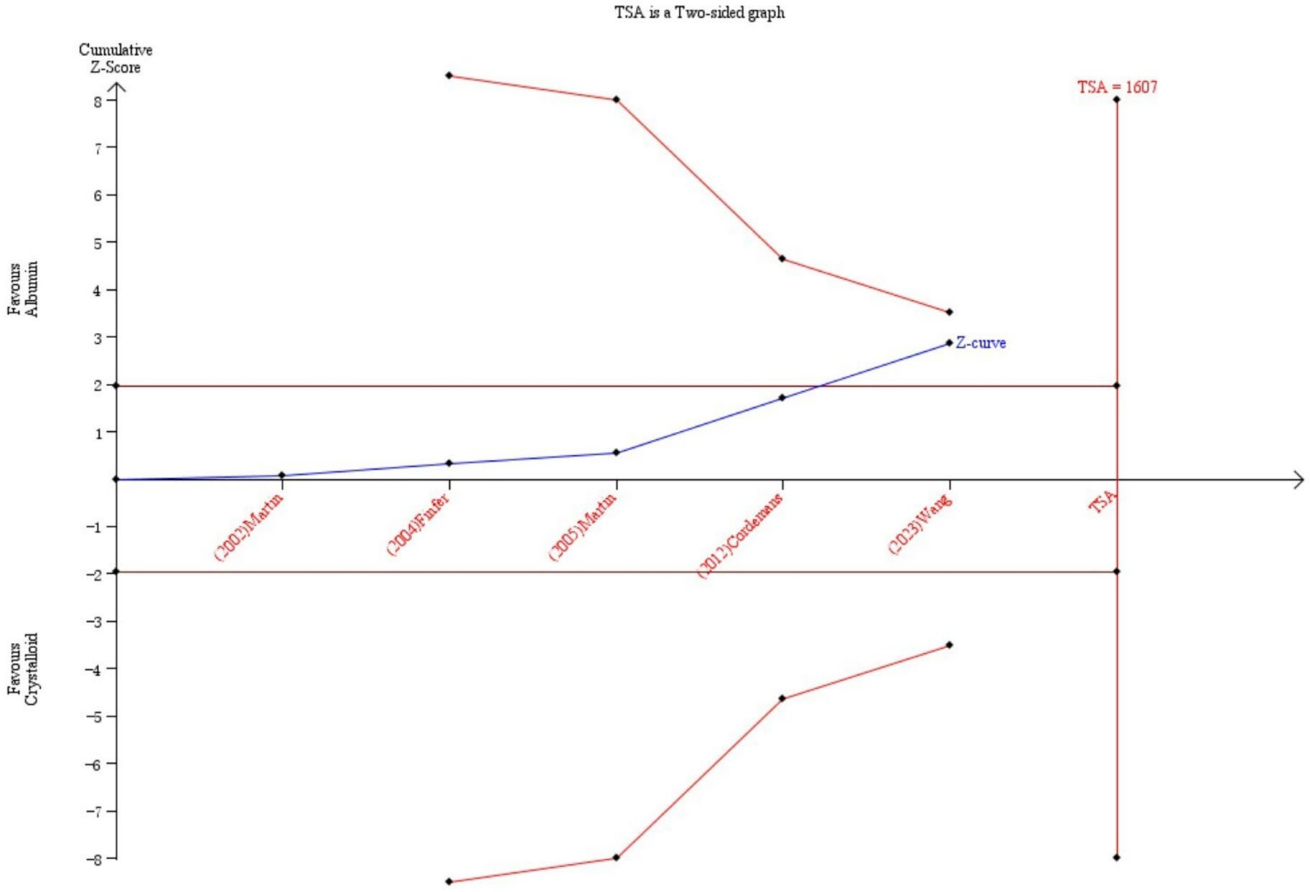
Trial sequential analysis of the effect of albumin on mortality in patients with ARDS. The cumulative Z-curve (solid line) tracks the accumulating evidence. The horizontal dashed lines represent the conventional boundaries for significance (Z = ±1.96). The inward-sloping curved lines are the trial sequential monitoring boundaries, adjusted for repeated testing. The vertical dashed line indicates the required information size (RIS) of 1,607 participants. As the Z-curve neither crosses the monitoring boundaries nor reaches the RIS, the analysis indicates that the cumulative evidence is insufficient to confirm or reject a 20% relative risk reduction in mortality with albumin therapy, underscoring the need for further trials.

TSA employed the Biggerstaff-Tweedie approach with these parameters: event proportion = 35.3% [3], α = 0.05, power = 80% (β = 0.20), and 20% relative risk reduction. This adjustment addressed random errors and repeated testing of limited data. The prespecified information size (1,607 participants) remained unmet (Figure 5). Since neither the monitoring boundary was crossed nor the required sample size achieved, the TSA suggested insufficient evidence to confirm albumin’s mortality benefit and high quality RCTs are still need.

## 4. Discussion

In this systematic review and meta-analysis, we found that albumin administration was associated with a significant reduction in 28-day mortality in adult patients with ARDS compared to crystalloid solutions. This potential benefit appeared to be driven by the use of hyper-oncotic albumin, as suggested by our subgroup analysis. Additionally, albumin therapy was associated with short-term improvements in oxygenation within the first 48 h, although this effect was not sustained beyond 72 h. It is crucial to interpret these findings in the context of the current evidence base and its limitations, as the trial sequential analysis indicated that the required information size has not yet been met, underscoring the need for further high-quality randomized controlled trials.

Patients with ARDS experience varying degrees of increased alveolar-capillary barrier permeability due to inflammation. Once the endothelial barrier is compromised, alveolar and interstitial pulmonary edema ensue, impairing gas exchange and leading to hypoxemia. Consequently, a conservative fluid management strategy is an integral part of ARDS treatment [3]. However, the systemic inflammatory response often results in a compromised endothelial barrier not limited to the lungs, also causing hypotension in a proportion of ARDS patients [5]. The optimal fluid management strategy must, therefore, effectively replenish circulating volume to ensure adequate oxygen delivery while avoiding worsening of pulmonary edema from excessive fluid administration [33].

Albumin, as a primary colloidal component in plasma, plays a crucial role in maintaining plasma oncotic pressure. The potential mortality benefit observed with hyper-oncotic albumin, as opposed to the neutral effect of iso-oncotic albumin, may be explained by mechanisms beyond simple volume expansion. Hyper-oncotic albumin, due to its high oncotic pressure, may more effectively draw fluid from the interstitial space, including the pulmonary interstitium, potentially reducing lung water more efficiently [19,20]. Furthermore, albumin possesses pleiotropic properties that may be beneficial in ARDS, including endothelial stabilization through glycocalyx protection, antioxidant effects, and anti-inflammatory properties [34]. These mechanisms might be particularly relevant in countering the core pathophysiology of ARDS and could explain the differential effect observed between albumin concentrations.

The studies included in this meta-analysis exhibit considerable clinical and methodological diversity, as detailed in Table 1. The interventions ranged from a targeted pharmaco-physiological approach to a pragmatic resuscitation fluid comparison and a real-world timing-based exposure. Rather than diminishing the value of our synthesis, this heterogeneity provides a broader, multi-faceted lens through which to examine the role of albumin in ARDS management. The fact that a signal toward reduced mortality consistently emerges across such varied contexts – spanning different study designs (prospective RCTs to retrospective analyses), patient populations (specific ALI/ARDS phenotypes to general critical illness), and intervention strategies (adjunctive hyperoncotic therapy to routine isotonic resuscitation) – lends a notable robustness to the central finding. It suggests that the potential benefit of albumin may not be confined to a single, highly specific clinical scenario but might be relevant across a spectrum of ARDS pathophysiology.

The challenge of pooling these varied interventions for a mortality outcome must be highlighted, which carries a risk of confounding the estimate. First, our analysis employed a random-effects model, which is more appropriate for heterogeneous studies as it does not assume a single true effect size and assigns more balanced weights. Second, we performed sensitivity analyses (e.g. sequential exclusion of each study, and analysis excluding the largest non-RCT cohort), which confirmed that the trend toward reduced mortality was not dependent on any single study design or population (Supplementary Material 5 Fig. S1). Third, and most importantly, the TSA indicated that the required information size has not been reached, which formally cautions against overinterpreting the nominal statistical significance of the pooled estimate. Therefore, while the consistency of the signal across diverse contexts is noteworthy, we present the pooled odds ratio not as a definitive measure of benefit, but as a robust statistical signal that strongly justifies the design of a future, definitive RCT with a homogeneous protocol.

Our findings contrast with a previous meta-analysis by Uhlig et al. [35], which included only RCTs and concluded that albumin may not reduce mortality. This discrepancy is likely attributable to our inclusion of non-randomized studies (NRSs), which significantly expanded the sample size from 204 to 588 patients and enhanced the statistical power to detect an effect. The mortality benefit was particularly pronounced in the NRSs (OR = 0.52, *p* = 0.002). However, this observation must be interpreted with considerable caution. The two included NRSs were assessed to have a moderate-to-high risk of bias using the ROBINS-I tool, particularly in the domains of selection and comparability. Therefore, the more favorable effect size in the NRSs likely overestimates the true treatment effect and must be interpreted as hypothesis-generating rather than conclusive.

Regarding oxygenation, our findings of early improvement are consistent with previous studies [12,14,31]. However, the clinical relevance of these statistically significant improvements must be considered alongside their transient nature, which is in line with observations from a recent meta-analysis on albumin-aided diuresis [36]. The lack of a sustained effect beyond 72 h and the absence of a significant reduction in ICU or hospital length of stay raise questions about the long-term clinical impact of these short-term physiological gains.

Our study has several limitations that should be considered when interpreting the results. Firstly, it only included 588 patients from 3 RCTs and 2 retrospective studies. To directly quantify this risk of random error and provide a more nuanced interpretation of our significant P-values, we preemptively conducted a TSA, which transparently highlights the need for further studies.

Second, to address the inherent limitation of a small number of RCTs and to enhance the statistical power of our analysis, we made the deliberate decision to include NRSs. While this strategy successfully expanded our sample size from 204 to 588 patients, it introduced a well-known potential for confounding and selection bias. To critically appraise this trade-off, we employed the ROBINS-I for quality assessment, which clearly identified methodological limitations in these studies, underscoring that the overall result should be interpreted with caution.

Third, we were unable to perform subgroup analyses based on ARDS etiology or severity due to the lack of reported data in the primary studies. Despite our best efforts to extract granular data, this was not possible. We have explicitly acknowledged this as a limitation to prevent over-interpretation of our results and to guide future research to address these clinically vital questions.

Finally, our promising subgroup analysis regarding hyper-oncotic albumin is based on only three studies and a small sample size, making it underpowered and exploratory in nature. We have been explicit in framing this finding as hypothesis-generating rather than confirmatory, and we have clearly reported the number of studies and patients in this subgroup to allow readers to judge the robustness for themselves.

## 5. Conclusion

This meta-analysis suggests that hyper-oncotic albumin may reduce mortality and improve early oxygenation in ARDS patients compared to crystalloids. However, the limited number of studies and inconclusive TSA indicate that current evidence is not definitive. Larger RCTs focusing on hyperoncotic albumin in ARDS are urgently needed to validate these findings and define its potential role in management.

## Supplementary Material

Supplementary Material5 neirong.docx

Supplementary Material4 paichu.docx

Supplementary Material3 jiansuocelue.docx

Supplementary Material1 PRISMA2020checklist .docx

Supplementary Material2 PRISMA2020abstract checklist.docx

## Data Availability

The data can be requested from the corresponding author
