# Supplementary material for: Hyper-oncotic albumin administration reduces mortality in acute Respiratory Distress Syndrome compared to crystalloid: a systematic review and meta-analysis
Source: Ann Med. 2026 Mar 24;58(1):2637271. doi: 10.1080/07853890.2026.2637271 (PMC13015065; doi:10.1080/07853890.2026.2637271)
Supplement: Supplementary Material5 neirong.docx [file IANN_A_2637271_SM5912.docx]

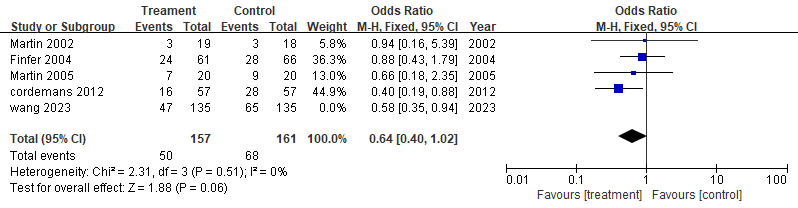


**Fig.S1** Sensitivity Analysis of the Effect of Albumin on Mortality in ARDS Patients.


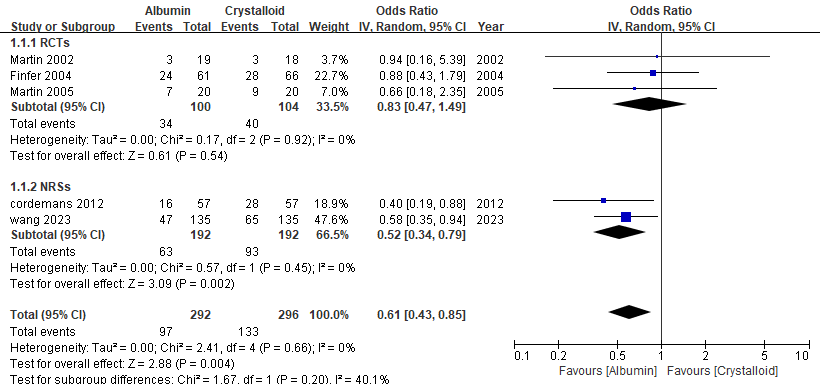


**Fig. S2** The effects of different types of research of albumin on reducing mortality in the treatment of ARDS

**
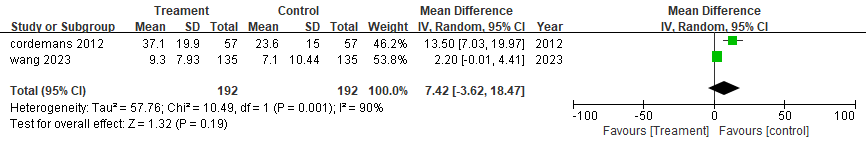
**

**Fig. S3** Impact of Albumin on Length of ICU Stay in ARDS Patients.


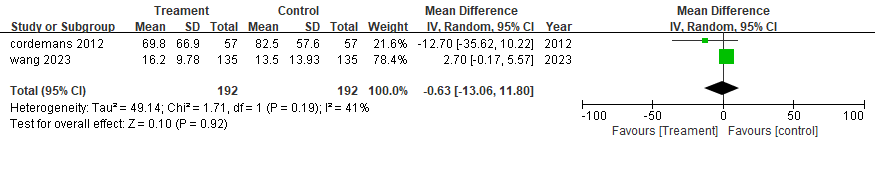


**Fig. S4** Impact of Albumin on Length of Hospital Stay in ARDS Patients.
