# Supplementary material for: Hyper-oncotic albumin administration reduces mortality in acute Respiratory Distress Syndrome compared to crystalloid: a systematic review and meta-analysis
Source: Ann Med. 2026 Mar 24;58(1):2637271. doi: 10.1080/07853890.2026.2637271 (PMC13015065; doi:10.1080/07853890.2026.2637271)
Supplement: Supplementary Material4 paichu.docx [file IANN_A_2637271_SM5911.docx]

**Additional File 2.** Excluded articles and reasons

**Excluded articles and reasons**

| **First author** | **Year** | **Title** | **Reason for exclusion** |
| --- | --- | --- | --- |
| Bland [1] | 1976 | Rapid infusion of sodium bicarbonate and albumin into high-risk premature infants soon after birth: a controlled, prospective trial. | 1. High-risk premature infants were included in the study; ARDS subgroup was not mentioned. |
| Lowe [2] | 1977 | Crystalloid vs colloid in the etiology of pulmonary failure after trauma: a randomized trial in man. | 1. Trauma patients were included in the study; ARDS subgroup was not mentioned. |
| Davies [3] | 1979 | South Wales multicenter trial of prophylactic Dextran 70 after surgery: a clinically oriented randomized double-blind trial. Further observations | 1. Patients after surgery were included in the study; ARDS subgroup was not mentioned. |
| Hauser [4] | 1980 | Oxygen transport responses to colloids and crystalloids in critically ill surgical patients. | 1.Mortality data was inadequate |
| Khosropour [5] | 1980 | Comparison of effects of hydroxyethyl starch (HES 200/0.5) administered pre- and postoperatively in vascular surgery with dextrane 40 (60) | 1. vascular surgery patients were included in the study; ARDS subgroup was not mentioned.  2. The study compared the difference between the hydroxyethyl starch solution and dextrane 40, without the comparison between albumin and crystalloid. |
| Lazrove [6] | 1980 | Hemodynamic, blood volume, and oxygen transport responses to albumin and hydroxyethyl starch infusions in critically ill postoperative patients | 1.Critically ill patients were included in the study; ARDS subgroup was not mentioned.  2.The study compared the difference between the hydroxyethyl starch solution and albumin, without the comparison between albumin and crystalloid. |
| Modig [7] | 1983 | Advantages of dextran 70 over Ringer acetate solution in shock treatment and in prevention of adult respiratory distress syndrome. A randomized study in man after traumatic-haemorrhagic shock | 1.Patients after traumatic-haemorrhagic shock were included in the study; ARDS subgroup was not mentioned.  2.The study compared the difference between the dextran 70 and Ringer acetate solution, without the comparison between albumin and crystalloid. |
| Metildi [8] | 1984 | Crystalloid versus colloid in fluid resuscitation of patients with severe pulmonary insufficiency | 1.Critically ill patients were included in the study; ARDS subgroup was not mentioned. |
| Reed [9] | 1985 | Dextran 70 versus donor plasma as colloid in open-heart surgery under extreme haemodilution. | 1. Open-heart surgery patients were included in the study; ARDS subgroup was not mentioned.  2.The study compared the difference between the dextran 70 and plasma, without the comparison between albumin and crystalloid. |
| Modig [10] | 1986 | Effectiveness of dextran 70 versus Ringer's acetate in traumatic shock and adult respiratory distress syndrome | 1. The study compared the difference between the dextran 70 and crystalloid, without the comparison between albumin and crystalloid. |
| Hankeln [11] | 1989 | Comparison of hydroxyethyl starch and lactacted Ringer's solution on hemodynamics and oxygen transport of critically ill patients in prospective crossover studies. | 1. The data of ARDS subgroup was inadequate (n=2).  2.The study compared the difference between the hydroxyethyl starch and crystalloid, without the comparison between albumin and crystalloid. |
| Foley [12] | 1990 | Albumin supplementation in the critically ill. A prospective, randomized trial. | 1.Critically ill patients were included in the study; ARDS subgroup was not mentioned. |
| Stockwell [13] | 1992 | Colloid solutions in the critically ill. A randomized comparison of albumin and polygeline. 1. Outcome and duration of stay in the intensive care unit. | 1.Critically ill patients were included in the study; ARDS subgroup was not mentioned. |
| Beards [14] | 1994 | Comparison of the hemodynamic and oxygen transport responses to modified fluid gelatin and hetastarch in critically ill patients: a prospective, randomized trial | 1.Critically ill patients were included in the study; ARDS subgroup was not mentioned.  2.The study compared the difference between the gelatin and hetastarch, without the comparison between albumin and crystalloid. |
| Bickell [15] | 1994 | Immediate versus delayed fluid resuscitation for hypotensive patients with penetrating torso injuries | 1. Trauma patients were included in the study; ARDS subgroup was not mentioned.  2.The study compared the difference between the immediate versus delayed fluid resuscitation, without the comparison between albumin and crystalloid. |
| Golub [16] | 1994 | Efficacy of albumin supplementation in the surgical intensive care unit: a prospective, randomized study | 1. Surgical patients were included in the study; ARDS subgroup was not mentioned. |
| Boldt [17] | 1995 | Does the type of volume therapy influence endothelial-related coagulation in the critically ill? | 1.Critically ill patients were included in the study; ARDS subgroup was not mentioned. |
| Yu [18] | 1995 | Frequency of mortality and myocardial infarction during maximizing oxygen delivery: a prospective, randomized trial. | 1.Critically ill patients were included in the study; ARDS subgroup was not mentioned. |
| Boldt [19] | 1996 | Influence of different volume therapy regimens on regulators of the circulation in the critically ill | 1.Critically ill patients were included in the study; ARDS subgroup was not mentioned. |
| Boldt [20] | 1996 | Influence of different volume therapies on platelet function in the critically ill | 1.Critically ill patients were included in the study; ARDS subgroup was not mentioned. |
| Boldt [21] | 1996 | The effects of albumin versus hydroxyethyl starch solution on cardiorespiratory and circulatory variables in critically ill patients | 1. Critically ill patients were included in the study; ARDS subgroup was not mentioned.  2.The study compared the difference between the hydroxyethyl starch solution and albumin without the comparison between albumin and crystalloid. |
| Wahba [22] | 1996 | Fluid resuscitation with Haemaccel (R) vs. human albumin following coronary artery bypass grafting | 1.Cardiac surgery patients were included in the study; ARDS subgroup was not mentioned. |
| Buhre [23] | 1997 | Acute effect of mitral calve replacement on extravascular lung water in patients receiving colloid or crystalloid priming of cardiopulmonary bypass | 1.Cardiac surgery patients were included in the study; ARDS subgroup was not mentioned. |
| Boldt [24] | 1998 | Volume therapy in the critically ill: is there a difference? | 1.Critically ill patients were included in the study; ARDS subgroup was not mentioned. |
| Ernest [25] | 1999 | Distribution of normal saline and 5% albumin infusions in septic patients. | 1.Septic patients were included in the study; ARDS subgroup was not mentioned. |
| Mbaba Mena [26] | 2000 | Effects of a hydroxyethylstarch solution on plasma colloid osmotic pressure in acutely ill patients. | 1. Critically ill patients were included in the study; ARDS subgroup was not mentioned. |
| Turner [27] | 2000 | A randomised controlled trial of prehospital intravenous fluid replacement therapy in serious trauma. | 1. Trauma patients were included in the study; ARDS subgroup was not mentioned. |
| Martin [28] | 2002 | Findings on the portable chest radiograph correlate with fluid balance in critically ill patients | 1.Mortality data was inadequate. |
| Neff [29] | 2003 | Repetitive large-dose infusion of the novel hydroxyethyl starch 130/0.4 in patients with severe head injury. | 1. Head injury patients were included in the study; ARDS subgroup was not mentioned.  2.The study compared the effect of the hydroxyethyl starch solution, without the comparison between albumin and crystalloid. |
| Molnár [30] | 2004 | Fluid resuscitation with colloids of different molecular weight in septic shock | 1. The study compared the difference between colloids of different molecular, without the comparison between albumin and crystalloid. |
| Quinlan [31] | 2004 | Albumin influences total plasma antioxidant capacity favorably in patients with acute lung injury | 1.Mortality data was inadequate. |
| Rittoo [32] | 2004 | Randomized study comparing the effects of hydroxyethyl starch solution with Gelofusine on pulmonary function in patients undergoing abdominal aortic aneurysm surgery. | 1. Patients undergoing abdominal aortic aneurysm surgery were included in the study; ARDS subgroup was not mentioned.  2.The study compared the effects of the hydroxyethyl starch solution, without the comparison between albumin and crystalloid. |
| Veneman [33] | 2004 | Human albumin and starch administration in critically ill patients: a prospective randomized clinical trial. | 1. Critically ill patients were included in the study; ARDS subgroup was not mentioned.  2.The study compared the difference between the hydroxyethyl starch solution and albumin without the comparison between albumin and crystalloid. |
| Bellomo [34] | 2006 | The effects of saline or albumin resuscitation on acid-base status and serum electrolytes | 1. Critically ill patients were included in the study; ARDS subgroup was not mentioned. |
| Bentsen [35] | 2006 | Hypertonic saline (7.2%) in 6% hydroxyethyl starch reduces intracranial pressure and improves hemodynamics in a placebo-controlled study involving stable patients with subarachnoid hemorrhage. | 1.Patients with subarachnoid hemorrhage were included in the study; ARDS subgroup was not mentioned.  2.The study compared the difference between the hypertonic saline in hydroxyethyl starch, without the comparison between albumin and crystalloid. |
| Bubois [36] | 2006 | Albumin administration improves organ function in critically ill hypoalbuminemic patients: A prospective, randomized, controlled, pilot study | 1. Critically ill patients were included in the study; ARDS subgroup was not mentioned.  2.mortality data of albumin group and saline group was inadequate |
| Moreau [37] | 2006 | Comparison of outcome in patients with cirrhosis and ascites following treatment with albumin or a synthetic colloid: A randomised controlled pilot trial. | 1. Patients with cirrhosis and ascites were included in the study; ARDS subgroup was not mentioned. |
| Palumbo [38] | 2006 | The effects of hydroxyethyl starch solution in critically ill patients. | 1. Critically ill patients were included in the study; ARDS subgroup was not mentioned.  2.The study compared the effects of the hydroxyethyl starch solution, without the comparison between albumin and crystalloid. |
| Verheij [39] | 2006 | Effect of fluid loading with saline or colloids on pulmonary permeability, oedema and lung injury score after cardiac and major vascular surgery. | 1. Patients after cardiac and major vascular surgery were included in the study; ARDS subgroup was not mentioned. |
| Krasheninnikov [40] | 2007 | Effect of various colloidal solutions on pulmonary oxygenizing function in patients with acute lung lesion | 1. Patients with acute lung lesion were included in the study; ARDS subgroup was not mentioned. |
| Kuper [41] | 2007 | The short-term effect of hyperoncotic albumin, given alone or with furosemide, on oxygenation in sepsis-induced acute respiratory distress syndrome. | 1.The study compared the difference between the albumin given alone and with furosemide, without the comparison between albumin and crystalloid. |
| Myburgh [42] | 2007 | Saline or albumin for fluid resuscitation in patients with traumatic brain injury. | 1. Patients with traumatic brain injury were included in the study; ARDS subgroup was not mentioned. |
| Bulger [43] | 2008 | Hypertonic resuscitation of hypovolemic shock after blunt trauma: a randomized controlled trial | 1. Hypovolemic shock patients were included in the study; ARDS subgroup was not mentioned. |
| Bellomo [44] | 2009 | Effects of saline or albumin resuscitation on standard coagulation tests | 1.Critically ill patients were included in the study; ARDS subgroup was not mentioned. |
| Dolecek [45] | 2009 | Therapeutic influence of 20 % albumin versus 6% hydroxyethyl starch on extravascular lung water in septic patients: a randomized controlled trial | 1. Septic patients were included in the study; ARDS subgroup was not mentioned.  2. The study compared the difference between the albumin and hydroxyethyl starch, without the comparison between albumin and crystalloid. |
| Heijden [46] | 2009 | Crystalloid or colloid fluid loading and pulmonary permeability, edema, and injury in septic and nonseptic critically ill patients with hypovolemia. | 1.Critically ill patients were included in the study; ARDS subgroup was not mentioned.  2.mortality data of albumin group and saline group was inadequate |
| Mahmood [47] | 2009 | Splanchnic Microcirculation Protection by Hydroxyethyl Starches During Abdominal Aortic Aneurysm Surgery. | 1. Abdominal aortic aneurysm surgery patients were included in the study; ARDS subgroup was not mentioned.  2. The study compared the effects of hydroxyethyl starch, without the comparison between albumin and crystalloid. |
| Senagore [48] | 2009 | Fluid management for laparoscopic colectomy: A prospective, randomized assessment of goal-directed administration of balanced salt solution or hetastarch coupled with an enhanced recovery program. | 1.Surgery patients were included in the study; ARDS subgroup was not mentioned. |
| Xie [49] | 2009 | Effect of continuous high-volume hemofiltration on patients with acute respiratory distress syndrome and multiple organ dysfunction syndrome | 1. The study focused the effects of continuous high-volume hemofiltration, without the comparison between albumin and crystalloid. |
| Forget [50] | 2010 | Goal-directed fluid management based on the pulse oximeter-derived pleth variability index reduces lactate levels and improves fluid management. | 1. Major abdominal surgery patients were included in the study; ARDS subgroup was not mentioned. |
| Gondos [51] | 2010 | Short-term effectiveness of different volume replacement therapies in postoperative hypovolaemic patients | 1. Postoperative hypovolaemic patients were included in the study; ARDS subgroup was not mentioned. |
| Trof [52] | 2010 | Greater cardiac response of colloid than saline fluid loading in septic and non-septic critically ill patients with clinical hypovolaemia. | 1.Critically ill patients were included in the study; ARDS subgroup was not mentioned. |
| Du [53] | 2011 | Hydroxyethyl starch resuscitation reduces the risk of intra-abdominal hypertension in severe acute pancreatitis. | 1. Severe acute pancreatitis patients were included in the study; ARDS subgroup was not mentioned.  2. The study compared the effects of hydroxyethyl starch, without the comparison between albumin and crystalloid. |
| Maitland [54] | 2011 | Mortality after fluid bolus in African children with severe infection. | 1.Sever infection patients were included in the study; data of ARDS was inadequate. |
| Yu [55] | 2011 | A prospective randomized trial using blood volume analysis in addition to pulmonary artery catheter, compared with pulmonary artery catheter alone, to guide shock resuscitation in critically ill surgical patients. | 1. The study compared the effects of measurement of blood volume, without the comparison between albumin and crystalloid. |
| Zhang [56] | 2011 | The effects of joint administration of 6% hydroxyethyl starch 130/0.4 and high-volume hemofiltration on patients with acute lung injury and acute kidney injury | 1. The study compared the effects of hydroxyethyl starch and high-volume hemofiltration, without the comparison between albumin and crystalloid. |
| Myburgh [57] | 2012 | Hydroxyethyl starch or saline for fluid resuscitation in intensive care | 1.Critically ill patients were included in the study; ARDS subgroup was not mentioned.  2. The study compared the differences between hydroxyethyl starch and saline, without the comparison between albumin and crystalloid. |
| Doungngern [58] | 2012 | Effect of albumin on diuretic response to furosemide in patients with hypoalbuminemia | 1.Critically ill patients were included in the study; ARDS subgroup was not mentioned.  2.Mortality data of albumin group and saline group was inadequate |
| Annane [59] | 2013 | Effects of fluid resuscitation with colloids vs crystalloids on mortality in critically ill patients presenting with hypovolemic shock: the CRISTAL randomized trial | 1.Hypovolemic shock patients were included in the study; data of ARDS was inadequate.  2.No comparison between albumin and crystalloid. |
| Chang [60] | 2014 | Volume of fluids administered during resuscitation for severe sepsis and septic shock and the development of the acute respiratory distress syndrome | 1.Septic shock patients were included in the study; ARDS subgroup was not mentioned. |
| Lammi [61] | 2015 | Response to fluid boluses in the fluid and catheter treatment trial | 1.The study focused on the hemodynamic responses of ARDS patients, without the comparison between albumin and crystalloid. |
| Smith [62] | 2015 | Hemodynamic and biochemical responses to fluid bolus therapy with human albumin solution, 4% versus 20%, in critically ill adults | 1.Critically ill patients were included in the study; ARDS subgroup was not mentioned.  2.The study compared the effect of different concentrations of albumin, without the comparison between albumin and crystalloid. |
| Spoelstra-de Man [63] | 2017 | Different effects of fluid loading with saline, gelatine, hydroxyethyl starch or albumin solutions on acid-base status in the critically ill | 1.Critically ill patients were included in the study; ARDS subgroup was not mentioned. |
| Fink [64] | 2018 | Cohort Study of Albumin versus Lactated Ringer's for Postoperative Cardiac Surgery Fluid Resuscitation in the Intensive Care Unit | 1.Postcardiac surgery patients were included in the study, ARDS subgroup was not mentioned. |
| Oczkowski [65] | 2018 | Furosemide and Albumin for Diuresis of Edema (FADE): A parallel-group, blinded, pilot randomized controlled trial | 1.Critically ill patients were included in the study; ARDS subgroup was not mentioned. |
| Xie [66] | 2020 | Clinical characteristics and outcomes of critically ill patients with novel coronavirus infectious disease (COVID-19) in China: a retrospective multicenter study | 1.Critical COVID-19 patients were included in the study; data of ARDS was inadequate.  2.This retrospective study compared serum albumin levels in patients with different prognoses, but did not compare albumin and crystalloid. |
| Mahmoodpoor [67] | 2020 | Efficacy of furosemide-albumin compared with furosemide in critically ill hypoalbuminemia patients admitted to intensive care unit: a prospective randomized clinical trial | 1.Critically ill hypoalbuminemia patients were included in the study; ARDS subgroup was not mentioned. |
| McNeil [68] | 2021 | Linear Association Between Hypoalbuminemia and Increased Risk of Acute Respiratory Distress Syndrome in Critically Ill Adults | 1.This study focused on the association between serum albumin levels and the occurrence of ARDS, did not comparison between albumin and crystalloid. |
| Gomez [69] | 2021 | Effects of 5% Albumin Plus Saline Versus Saline Alone on Outcomes From Large-Volume Resuscitation in Critically Ill Patients | 1.Critically ill patients were included in the study; ARDS subgroup was not mentioned. |
| Côté [70] | 2021 | Diuretic strategies in patients with resistance to loop-diuretics in the intensive care unit: A retrospective study from the MIMIC-III database | 1.Critically ill patients were included in the study; ARDS subgroup was not mentioned.  2.Mortality data of albumin group and saline group was inadequate |
| Zhang [71] | 2022 | Albumin Infusion May Improve the Prognosis of Critical COVID-19 Patients with Hypoalbuminemia in the Intensive Care Unit: A Retrospective Cohort Study | 1.Critical COVID-19 patients were included in the study; data of ARDS was inadequate. |
| Gelbart [72] | 2022 | Characteristics and Physiologic Changes After 4% Albumin Fluid Boluses in a PICU | 1.Children in a cardiac and general PICU were included in the study, ARDS subgroup was not mentioned. |
| Abdelhamid [73] | 2024 | The Effect of Albumin Administration in Critically Ill Patients: A Retrospective Single-Center Analysis | 1.Critically ill patients were included in the study; ARDS subgroup was not mentioned. |
| Lu [74] | 2024 | Identifying new safety risk of human serum albumin: a retrospective study of real-world data | 1.The patients in FAERS database were included in the study, ARDS subgroup was not mentioned. |
| Qin [75] | 2024 | Early high-volume resuscitation with crystalloid solution combined with albumin improves survival of critically ill patients: A retrospective analysis from MIMIC-IV database | 1.Critically ill patients were included in the study; ARDS subgroup was not mentioned. |
| Rabi [76] | 2024 | The role of serum albumin in critical illness, predicting poor outcomes, and exploring the therapeutic potential of albumin supplementation | 1.Critically ill patients were included in the study; ARDS subgroup was not mentioned. |
| Gray [77] | 2024 | Albumin Versus Balanced Crystalloid for the Early Resuscitation of Sepsis: An Open Parallel-Group Randomized Feasibility Trial- The ABC-Sepsis Trial | 1.Sepsis patients were included in the study; ARDS subgroup was not mentioned. |

**References**

1. Bland RD, Clarke TL, Harden LB. Rapid infusion of sodium bicarbonate and albumin into high-risk premature infants soon after birth: a controlled, prospective trial. Am J Obstet Gynecol. 1976;124(3):263-267.
2. Lowe RJ, Moss GS, Jilek J, et al. Crystalloid vs colloid in the etiology of pulmonary failure after trauma: a randomized trial in man. Surgery. 1977;81(6):676-683.
3. Davies WT. South Wales multicenter trial of prophylactic Dextran 70 after surgery: a clinically oriented randomized double-blind trial. Further observations. Thromb Haemost. 1979;41(1):83-89.
4. Hauser CJ, Shoemaker WC, Turpin I, et al. Oxygen transport responses to colloids and crystalloids in critically ill surgical patients. Surg Gynecol Obstet. 1980;150(6):811-816.
5. Khosropour R, Lackner F, Steinbereithner K, et al. Comparison of effects of hydroxyethylstarch (HES 200/0.5) administered pre- and postoperatively in vascular surgery with dextran 40 (60) (author's transl). Anaesthesist 1980, 29(11):616-622
6. Lazrove S, Waxman K, Shippy C, et al. Hemodynamic, blood volume, and oxygen transport responses to albumin and hydroxyethyl starch infusions in critically ill postoperative patients. Crit Care Med. 1980;8(5):302-306.
7. Modig J. Advantages of dextran 70 over Ringer acetate solution in shock treatment and in prevention of adult respiratory distress syndrome. A randomized study in man after traumatic-haemorrhagic shock. Resuscitation. 1983;10(4):219-226.
8. Metildi LA, Shackford SR, Virgilio RW, et al. Crystalloid versus colloid in fluid resuscitation of patients with severe pulmonary insufficiency. Surg Gynecol Obstet. 1984;158(3):207-212.
9. Reed RK, Lilleaasen P, Lindberg H, et al. Dextran 70 versus donor plasma as colloid in open-heart surgery under extreme haemodilution. Scand J Clin Lab Invest. 1985;45(3):269-274.
10. Modig J. Effectiveness of dextran 70 versus Ringer's acetate in traumatic shock and adult respiratory distress syndrome. Crit Care Med. 1986;14(5):454-457.
11. Hankeln K, Rädel C, Beez M, et al. Comparison of hydroxyethyl starch and lactated Ringer's solution on hemodynamics and oxygen transport of critically ill patients in prospective crossover studies. Crit Care Med. 1989;17(2):133-135.
12. Foley EF, Borlase BC, Dzik WH, et al. Albumin supplementation in the critically ill. A prospective, randomized trial. Arch Surg. 1990;125(6):739-742.
13. Stockwell MA, Soni N, Riley B. Colloid solutions in the critically ill. A randomised comparison of albumin and polygeline. 1. Outcome and duration of stay in the intensive care unit. Anaesthesia. 1992;47(1):3-6.
14. Beards SC, Watt T, Edwards JD, et al. Comparison of the hemodynamic and oxygen transport responses to modified fluid gelatin and hetastarch in critically ill patients: a prospective, randomized trial. Crit Care Med. 1994;22(4):600-605.
15. Bickell WH, Wall MJ Jr, Pepe PE, et al. Immediate versus delayed fluid resuscitation for hypotensive patients with penetrating torso injuries. N Engl J Med. 1994;331(17):1105-1109.
16. Golub R, Sorrento JJ Jr, Cantu R Jr, et al. Efficacy of albumin supplementation in the surgical intensive care unit: a prospective, randomized study. Crit Care Med. 1994;22(4):613-619.
17. Boldt J, Heesen M, Welters I, et al. Does the type of volume therapy influence endothelial-related coagulation in the critically ill? Br J Anaesth. 1995;75(6):740-746.
18. Yu M, Takanishi D, Myers SA, et al. Frequency of mortality and myocardial infarction during maximizing oxygen delivery: a prospective, randomized trial. Crit Care Med. 1995;23(6):1025-1032.
19. Boldt J, Mueller M, Menges T, et al. Influence of different volume therapy regimens on regulators of the circulation in the critically ill. Br J Anaesth. 1996;77(4):480-487.
20. Boldt J, Müller M, Heesen M, et al. Influence of different volume therapies on platelet function in the critically ill. Intensive Care Med. 1996;22(10):1075-1081.
21. Boldt J, Heesen M, Müller M, et al. The effects of albumin versus hydroxyethyl starch solution on cardiorespiratory and circulatory variables in critically ill patients. Anesth Analg. 1996;83(2):254-261.
22. Wahba A, Sendtner E, Birnbaum DE. Fluid resuscitation with Haemaccel vs. human albumin following coronary artery bypass grafting. Thorac Cardiovasc Surg. 1996;44(4):178-182.
23. Buhre W, Hoeft A, Schorn B, et al. Acute effect of mitral calve replacement on extravascular lung water in patients receiving colloid or crystalloid priming of cardiopulmonary bypass. Br J Anaesth 1997, 79(3):311-316
24. Boldt J, Müller M, Mentges D, et al. Volume therapy in the critically ill: is there a difference? Intensive Care Med. 1998;24(1):28-36.
25. Ernest D, Belzberg AS, Dodek PM. Distribution of normal saline and 5% albumin infusions in septic patients. Crit Care Med. 1999;27(1):46-50.
26. Mbaba Mena J, De Backer D, Vincent JL. Effects of a hydroxyethylstarch solution on plasma colloid osmotic pressure in acutely ill patients. Acta Anaesthesiol Belg. 2000;51(1):39-42.
27. Turner J, Nicholl J, Webber L, et al. A randomised controlled trial of prehospital intravenous fluid replacement therapy in serious trauma. Health Technol Assess. 2000;4(31):1-57.
28. Martin GS, Ely EW, Carroll FE, et al. Findings on the portable chest radiograph correlate with fluid balance in critically ill patients. Chest. 2002;122(6):2087-2095.
29. Neff TA, Doelberg M, Jungheinrich C, et al. Repetitive large-dose infusion of the novel hydroxyethyl starch 130/0.4 in patients with severe head injury. Anesth Analg. 2003;96(5):1453-1459.
30. Molnár Z, Mikor A, Leiner T, et al. Fluid resuscitation with colloids of different molecular weight in septic shock. Intensive Care Med. 2004;30(7):1356-1360.
31. Quinlan GJ, Mumby S, Martin GS, et al. Albumin influences total plasma antioxidant capacity favorably in patients with acute lung injury. Crit Care Med. 2004;32(3):755-759.
32. Rittoo D, Gosling P, Burnley S, et al. Randomized study comparing the effects of hydroxyethyl starch solution with Gelofusine on pulmonary function in patients undergoing abdominal aortic aneurysm surgery. Br J Anaesth. 2004;92(1):61-66.
33. Veneman TF, Oude Nijhuis J, Woittiez AJ. Human albumin and starch administration in critically ill patients: a prospective randomized clinical trial. Wien Klin Wochenschr. 2004;116(9-10):305-309.
34. Bellomo R, Morimatsu H, French C, et al. The effects of saline or albumin resuscitation on acid-base status and serum electrolytes. Crit Care Med. 2006;34(12):2891-2897.
35. Bentsen G, Breivik H, Lundar T, et al. Hypertonic saline (7.2%) in 6% hydroxyethyl starch reduces intracranial pressure and improves hemodynamics in a placebo-controlled study involving stable patients with subarachnoid hemorrhage. Crit Care Med. 2006;34(12):2912-2917.
36. Dubois MJ, Orellana-Jimenez C, Melot C, et al. Albumin administration improves organ function in critically ill hypoalbuminemic patients: A prospective, randomized, controlled, pilot study. Crit Care Med. 2006;34(10):2536-2540.
37. Moreau R, Valla DC, Durand-Zaleski I, et al. Comparison of outcome in patients with cirrhosis and ascites following treatment with albumin or a synthetic colloid: a randomised controlled pilot trail. Liver Int. 2006;26(1):46-54.
38. Palumbo D, Servillo G, D'Amato L, et al. The effects of hydroxyethyl starch solution in critically ill patients. Minerva Anestesiol. 2006;72(7-8):655-664.
39. Verheij J, van Lingen A, Raijmakers PG, et al. Effect of fluid loading with saline or colloids on pulmonary permeability, oedema and lung injury score after cardiac and major vascular surgery. Br J Anaesth. 2006;96(1):21-30.
40. Krasheninnikov SV, Levit AL, Leĭderman IN, et al. Effect of various colloidal solutions on pulmonary oxygenizing function in patients with acute lung lesion. Anesteziol Reanimatol. 2007;(3):20-22.
41. Kuper M, Gunning MP, Halder S, et al. The short-term effect of hyperoncotic albumin, given alone or with furosemide, on oxygenation in sepsis-induced acute respiratory distress syndrome. Anaesthesia. 2007;62(3):259-263.
42. SAFE Study Investigators, Australian and New Zealand Intensive Care Society Clinical Trials Group, Australian Red Cross Blood Service, et al. Saline or albumin for fluid resuscitation in patients with traumatic brain injury. The New England journal of medicine, 357(9), 874–884.
43. Bulger EM, Jurkovich GJ, Nathens AB, et al. Hypertonic resuscitation of hypovolemic shock after blunt trauma: a randomized controlled trial. Arch Surg. 2008;143(2):139-149.
44. Bellomo, R., Morimatsu, H., Presneill, J., et al. Effects of saline or albumin resuscitation on standard coagulation tests. Critical care and resuscitation : journal of the Australasian Academy of Critical Care Medicine, 11(4), 250–256.
45. Dolecek, M., Svoboda, P., Kantorová, I., et al. Therapeutic influence of 20 % albumin versus 6% hydroxyethylstarch on extravascular lung water in septic patients: a randomized controlled trial. Hepato-gastroenterology, 56(96), 1622–1628.
46. van der Heijden M, Verheij J, van Nieuw Amerongen GP, et al. Crystalloid or colloid fluid loading and pulmonary permeability, edema, and injury in septic and nonseptic critically ill patients with hypovolemia. Crit Care Med. 2009;37(4):1275-1281.
47. Mahmood A, Gosling P, Barclay R, et al. Splanchnic microcirculation protection by hydroxyethyl starches during abdominal aortic aneurysm surgery. Eur J Vasc Endovasc Surg. 2009;37(3):319-325.
48. Senagore AJ, Emery T, Luchtefeld M, et al. Fluid management for laparoscopic colectomy: a prospective, randomized assessment of goal-directed administration of balanced salt solution or hetastarch coupled with an enhanced recovery program. Dis Colon Rectum. 2009;52(12):1935-1940.
49. Xie J, Yang J. Effect of continuous high-volume hemofiltration on patients with acute respiratory distress syndrome and multiple organ dysfunction syndrome. Zhongguo Wei Zhong Bing Ji Jiu Yi Xue. 2009;21(7):402-404.
50. Forget P, Lois F, de Kock M. Goal-directed fluid management based on the pulse oximeter-derived pleth variability index reduces lactate levels and improves fluid management. Anesth Analg. 2010;111(4):910-914.
51. Gondos T, Marjanek Z, Ulakcsai Z, et al. Short-term effectiveness of different volume replacement therapies in postoperative hypovolaemic patients. Eur J Anaesthesiol. 2010;27(9):794-800.
52. Trof RJ, Sukul SP, Twisk JW, et al. Greater cardiac response of colloid than saline fluid loading in septic and non-septic critically ill patients with clinical hypovolaemia. Intensive Care Med. 2010;36(4):697-701.
53. Du XJ, Hu WM, Xia Q, et al. Hydroxyethyl starch resuscitation reduces the risk of intra-abdominal hypertension in severe acute pancreatitis. Pancreas. 2011;40(8):1220-1225.
54. Maitland K, Kiguli S, Opoka RO, et al. Mortality after fluid bolus in African children with severe infection. N Engl J Med. 2011;364(26):2483-2495.
55. Yu M, Pei K, Moran S, et al. A prospective randomized trial using blood volume analysis in addition to pulmonary artery catheter, compared with pulmonary artery catheter alone, to guide shock resuscitation in critically ill surgical patients. Shock. 2011;35(3):220-228.
56. Zhang JC, Ren HS, Jiang JJ, et al. The effects of joint administration of 6% hydroxyethyl starch 130/0.4 and high-volume hemofiltration on patients with acute lung injury and acute kidney injury. Zhongguo Wei Zhong Bing Ji Jiu Yi Xue. 2011;23(12):755-758.
57. Myburgh JA, Finfer S, Bellomo R, et al. Hydroxyethyl starch or saline for fluid resuscitation in intensive care [published correction appears in N Engl J Med. 2016 Mar 31;374(13):1298. doi: 10.1056/NEJMx160007]. N Engl J Med. 2012;367(20):1901-1911.
58. Doungngern T, Huckleberry Y, Bloom JW, et al. Effect of albumin on diuretic response to furosemide in patients with hypoalbuminemia. Am J Crit Care. 2012;21(4):280-286.
59. Annane D, Siami S, Jaber S, et al. Effects of fluid resuscitation with colloids vs crystalloids on mortality in critically ill patients presenting with hypovolemic shock: the CRISTAL randomized trial [published correction appears in JAMA. 2013 Mar 12;311(10):1071. Régnier, Jean [corrected to Reignier, Jean]; Cle'h, Christophe [corrected to Clec'h, Christophe]]. JAMA. 2013;310(17):1809-1817.
60. Chang DW, Huynh R, Sandoval E, et al. Volume of fluids administered during resuscitation for severe sepsis and septic shock and the development of the acute respiratory distress syndrome. J Crit Care. 2014;29(6):1011-1015.
61. Lammi MR, Aiello B, Burg GT, et al. Response to fluid boluses in the fluid and catheter treatment trial. Chest. 2015;148(4):919-926.
62. Bannard-Smith J, Alexander P, Glassford N, et al. Haemodynamic and biochemical responses to fluid bolus therapy with human albumin solution, 4% versus 20%, in critically ill adults. Crit Care Resusc. 2015;17(2):122-128.
63. Spoelstra-de Man AM, Smorenberg A, Groeneveld AB. Different effects of fluid loading with saline, gelatine, hydroxyethyl starch or albumin solutions on acid-base status in the critically ill. PLoS One. 2017;12(4):e0174507.
64. Fink RJ, Young A, Yanez ND, et al. Cohort Study of Albumin versus Lactated Ringer's for Postoperative Cardiac Surgery Fluid Resuscitation in the Intensive Care Unit. Pharmacotherapy. 2018;38(12):1241-1249.
65. Oczkowski SJW, Klotz L, Mazzetti I, et al. Furosemide and Albumin for Diuresis of Edema (FADE): A parallel-group, blinded, pilot randomized controlled trial. J Crit Care. 2018;48:462-467.
66. Xie J, Wu W, Li S, et al. Clinical characteristics and outcomes of critically ill patients with novel coronavirus infectious disease (COVID-19) in China: a retrospective multicenter study. Intensive Care Med. 2020;46(10):1863-1872.
67. Mahmoodpoor A, Zahedi S, Pourakbar A, et al. Efficacy of furosemide-albumin compared with furosemide in critically ill hypoalbuminemia patients admitted to intensive care unit: a prospective randomized clinical trial. Daru. 2020;28(1):263-269.
68. McNeil JB, Jackson KE, Wang C, et al. Linear Association Between Hypoalbuminemia and Increased Risk of Acute Respiratory Distress Syndrome in Critically Ill Adults. Crit Care Explor. 2021;3(9):e0527.
69. Gomez H, Priyanka P, Bataineh A, et al. Effects of 5% Albumin Plus Saline Versus Saline Alone on Outcomes From Large-Volume Resuscitation in Critically Ill Patients. Crit Care Med. 2021;49(1):79-90.
70. Côté JM, Bouchard J, Murray PT, et al. Diuretic strategies in patients with resistance to loop-diuretics in the intensive care unit: A retrospective study from the MIMIC-III database. J Crit Care. 2021;65:282-291.
71. Zhang L, Yu W, Zhao Y, et al. Albumin Infusion May Improve the Prognosis of Critical COVID-19 Patients with Hypoalbuminemia in the Intensive Care Unit: A Retrospective Cohort Study. Infect Drug Resist. 2022;15:6039-6050.
72. Gelbart B, Fulkoski N, Stephens D, et al. Characteristics and Physiologic Changes After 4% Albumin Fluid Boluses in a PICU. Pediatr Crit Care Med. 2022;23(1):e10-e19.
73. Abdelhamid S, Achermann R, Hollinger A, et al. The Effect of Albumin Administration in Critically Ill Patients: A Retrospective Single-Center Analysis. Crit Care Med. 2024;52(5):e234-e244.
74. Lu H, Zhang Y, Liu P. Identifying new safety risk of human serum albumin: a retrospective study of real-world data. Front Pharmacol. 2024;15:1319900.
75. Yalan Q, Yinzhou L, Binfei T, et al. Early high-volume resuscitation with crystalloid solution combined with albumin improves survival of critically ill patients: A retrospective analysis from MIMIC-IV database. Burns. 2024;50(4):893-902.
76. Rabi R, Alsaid RM, Matar AN, et al. The role of serum albumin in critical illness, predicting poor outcomes, and exploring the therapeutic potential of albumin supplementation. Sci Prog. 2024;107(3):368504241274023.
77. Gray AJ, Oatey K, Grahamslaw J, et al. Albumin Versus Balanced Crystalloid for the Early Resuscitation of Sepsis: An Open Parallel-Group Randomized Feasibility Trial- The ABC-Sepsis Trial. Crit Care Med. 2024;52(10):1520-1532.
