# Supplementary material for: Hyper-oncotic albumin administration reduces mortality in acute Respiratory Distress Syndrome compared to crystalloid: a systematic review and meta-analysis
Source: Ann Med. 2026 Mar 24;58(1):2637271. doi: 10.1080/07853890.2026.2637271 (PMC13015065; doi:10.1080/07853890.2026.2637271)
Supplement: Supplementary Material3 jiansuocelue.docx [file IANN_A_2637271_SM5910.docx]

**Additional File 1.** Search strategy

**Database: Pubmed**

------------------------------------------------------------------------

((albumin) OR (human serum albumin) OR (human albumin) OR (albumin replaement) OR (colloid) OR (crystalloid) OR (crystalloid solution)) AND ((acute respiratory distress syndrome) OR (ARDS) OR (acute lung injury) OR (ALI) OR (critical ill) OR (intensive care unit) OR (critical care) OR (ICU) OR (intensive care)) AND ((randomized controlled trial[pt]) OR (controlled clinical trial[pt]) OR (placebo[tiab]) OR (retrospective[tiab]) OR (random*[tiab] AND (trial[tiab] OR study[tiab] OR group[tiab])) OR (("controlled"[tiab] OR "comparative"[tiab]) AND ("trial"[tiab] OR "study"[tiab]))) / (5,465)

**Database: Science Direct**

--------------------------------------------------------------------------------

1. ((human serum albumin) OR (colloid)) AND ((acute respiratory distress syndrome) OR (acute lung injury)) AND ((randomized controlled trial) OR (controlled clinical trial)) / (17738)

2. Limit 1 to research articles (5704)

**Database: The Cochrane Controlled Trials Register**

--------------------------------------------------------------------------------

((albumin) OR (human serum albumin) OR (human albumin) OR (albumin replaement) OR (colloid) OR (crystalloid) OR (crystalloid solution)) AND ((acute respiratory distress syndrome) OR (ARDS) OR (acute lung injury) OR (ALI) OR (critical ill) OR (intensive care unit) OR (critical care) OR (ICU) OR (intensive care)) AND ((randomized controlled trial) OR (controlled clinical trial) OR (placebo) OR (retrospective) OR (random* AND (trial OR study OR group)) OR ((controlled OR comparative) AND (trial OR study)))/ (1811)

**Database:** **Web of Science**

--------------------------------------------------------------------------------

#1 (TS=albumin) OR (TS=human serum albumin) OR (TS=human albumin) OR (TS=albumin replaement) OR (TS=colloid) OR (TS=crystalloid) OR (TS=crystalloid solution) / (947,460)

#2 (TS=acute respiratory distress syndrome) OR (TS=ARDS) OR (TS=acute lung injury) OR (TS=ALI) OR (TS=critical ill) OR (TS=intensive care unit) OR (TS=critical care) OR (TS=ICU) OR (TS=intensive care) / (881,466)

#3 (TI=randomized controlled trial) OR (TI=controlled clinical trial) OR (TI=placebo) OR (TI=retrospective) / (1,159,735)

#1 AND #2 AND #3 / (897)

**Database: Scopus**

--------------------------------------------------------------------------------

( TITLE ( ( "albumin*" ) OR ( "human serum albumin" ) OR ( "human albumin" ) OR ( "albumin replaement" ) OR ( colloid ) OR ( crystalloid ) OR ( "crystalloid solution" ) ) AND TITLE-ABS-KEY ( ( "acute respiratory distress syndrome" ) OR ( ards ) OR ( "acute lung injury" ) OR ( ali ) OR ( "critical ill" ) OR ( "intensive care unit" ) OR ( "critical care" ) OR ( icu ) OR ( "intensive care" ) ) AND TITLE-ABS-KEY ( ( "randomized controlled trial" ) OR ( "controlled clinical trial" ) OR ( ( random* ) AND ( ( trial ) OR ( study ) OR ( group ) ) ) OR ( ( ( controlled ) OR ( comparative ) ) AND ( ( trial ) OR ( study ) ) ) OR ( placebo ) OR ( retrospective ) ) )/ (1032)
